# Establishing thresholds for swing transparency at the knee during gait to inform exoskeleton design

**DOI:** 10.1371/journal.pone.0317259

**Published:** 2025-01-17

**Authors:** Chase W. Mathews, Delaney A. Clawson, Karl E. Zelik

**Affiliations:** 1 Department of Mechanical Engineering, Vanderbilt University, Nashville, TN, United States of America; 2 Department of Biomedical Engineering, Vanderbilt University, Nashville, TN, United States of America; 3 Department of Physical Medicine and Rehabilitation, Vanderbilt University, Nashville, TN, United States of America; Polytechnic University of Marche: Universita Politecnica delle Marche, ITALY

## Abstract

Knee exoskeletons have been developed to assist, stabilize, or improve human movement or recovery. However, exoskeleton designers must implement transparency (i.e., get out of the way) modes during the swing phase of locomotor tasks to avoid impeding movement. The problem is that it is not understood how sensitive people are to small knee torques or what level of knee impedance is acceptable (sufficiently transparent) during swing phase. Here, we (i) characterized the biomechanical consequences of knee stiffness and damping during swing, and (ii) leveraged user perceptions of being impeded and toe clearance to define transparency thresholds, below which the participants were sufficiently unimpeded during the swing phase of gait. We conducted a series of human subject experiments that involved walking and stair ascent/descent while wearing a modified knee brace with five stiffness values ranging from 0 to 4 Nm/rad and five damping values ranging from 0 to 0.77 Nm/rad/s. We measured changes to lower limb kinematics, knee flexor muscle activity, and participants’ perception of being impeded during swing. Kinematics, muscle activity, and perceived impedance all changed in response to added stiffness and damping. For stiffness, we found the median transparency thresholds for walking and stairs to be 1.76 Nm/rad and 2.95 Nm/rad, respectively, which corresponds to peak knee moments during swing of around 2.3 and 5 Nm. For damping, we found the median transparency threshold for walking and stairs to be about the same, 0.29 Nm/rad/s, which corresponds to peak knee moments during swing of around 2.3 Nm. These values provide useful benchmarks for defining quantitative design requirements for knee exoskeletons intended for locomotor activities.

## Introduction

Knee exoskeletons have been developed for a wide range of locomotor activities including gait rehabilitation [1, 2], injury prevention [3], performance augmentation [4], and energy harvesting [5]. However, it is crucial for designers to consider the interaction between the device and the user during the entire gait cycle. In stance phase, the muscles about the knee generate large torques to support the body [6]. Designers of knee exoskeletons commonly aim to assist, resist, or augment the knee during portions of stance phase. In swing phase, knee joint torques are generally low as the joint undergoes large flexion and extension angles to clear the ground and position the foot for the next step [7]. Often designers aim to be transparent (i.e., get out of the way) during swing phase since the leg is especially prone to being perturbed or impeded due to the low-torque and precise motion. Wearable devices such as exoskeletons or knee braces must be carefully designed not to restrict knee motion during swing phase. Impeding the knee may cause user discomfort, aggravation, or maladaptive effects such as hip hiking, circumduction, foot scuffing, or stumbling, which can lead to higher joint loading or fall risk [8].

Exoskeleton developers often implement a *transparency* mode during swing phase. Transparency mode is a state during which the exoskeleton is supposed to behave *transparently*; that is, the exoskeleton does not alter the user’s joint trajectories, patterns of muscle activation, or subjective feelings of being impeded. There are a couple of common implementations, depending on the hardware design. For example, in high-torque robotic systems where there exists significant backdrive torque when the motor is simply left unpowered, an actively controlled transparency mode can be implemented. This controller can use force-feedback to try regulate the output force to be close to zero, or it may set virtual stiffness and damping values [9] to achieve the desired swing phase behavior. In passive mechanical exoskeletons, such as knee extension-assist exoskeletons [10], swing-phase transparency can be achieved by physically disengaging a spring [11, 12], or by changing the set point or stiffness of a spring acting about the knee. However, even during transparency mode, powered and passive exoskeletons still apply small (non-zero) torques about the knee from various sources such as friction, springs, gears, backlash, control errors, imperfect sensing, or maintaining lightly tensioned cables [13, 14]. However, it is not well understood how sensitive people are to these small torques, nor the acceptable upper limit of torques applied to the knee during swing. Thus, there exists a key knowledge gap: there is currently no objective or engineering threshold defining what it actually means for a knee exoskeleton to be transparent.

One common way designers have tried to demonstrate the efficacy of transparency mode is by measuring swing-phase gait kinematics with the exoskeleton on (in transparency mode) and without the exoskeleton. If no significant changes are found between the kinematics (e.g., knee flexion range of motion), then the exoskeleton is considered to be adequately transparent [15]. However, these tests are generally performed during a single speed of walking and not across multiple locomotion tasks, such as stair ascent. Furthermore, while maintaining kinematics is important for transparency, it does not provide a complete picture of whether (or not) swing phase is impeded [16]. Humans adapt to changes in lower limb impedance [17]–for example, after subjecting one limb to a higher inertia, participants adjusted their kinematics in response to the new lower-limb properties within 45 to 50 strides [18]. Coifman et al. reported that adding a 1 kg distal mass during running only increased knee flexion angle by 6%, but increased joint moment and power by 40 and 50%, respectively [19]. Taking into account neuromechanical adaptations, significant increases in muscle activity may also indicate an impeded swing phase despite retaining normal kinematics. Finally, independent of kinematics or muscle effort, the subjective perception of being impeded is also an important metric to consider when evaluating transparency and the overall impact and usability of wearable devices. The perception of being impeded by an exoskeleton is multi-factorial and may depend on user discomfort [20] or a feeling of reduced volition as natural motion is interfered or obstructed. Thus, open questions remain related to swing phase transparency when wearing exoskeletons.

The objective of this study was to establish transparency thresholds for knee exoskeletons during the swing phase of walking in order to inform device design. We pursued this by quantifying how different knee joint stiffness and damping parameters affected gait biomechanics (e.g., knee flexion and hamstring muscle activity) and subjective perception of being impeded. The quantified biomechanical and perceptual responses were used to provide a baseline level of stiffness, damping, and moment at the knee at which user experience was affected.

## Materials and methods

In this section, we describe an experiment to investigate the biomechanical and perceived consequences of exoskeleton stiffness and damping at the knee. We overview (i) participant recruitment and selection, (ii) modifications to a knee brace to enable reconfigurable damping and stiffness properties, (iii) data collection and processing, and (iv) execution of the experimental protocol.

### Participants

We recruited 10 healthy young participants without any knee injuries or diseases to participate in the study. Participants were excluded from participating in the study if the brace did not fit comfortably or if the brace significantly migrated on the leg during walking. The participant group consisted of a convenience sample of 5 males and 5 females with mean age of 24.3 ± 2.8 years, mean height of 178 ± 7.0 cm, and mean body mass of 72.6 ± 7.0 kg. The recruitment period started on January 1, 2024 and ended on April 1, 2024. All experimental procedures were approved by Vanderbilt University’s Institutional Review Board (IRB # 141697), and all participants provided written informed consent to participate in the study.

### Modified knee brace

**Fig 1:**
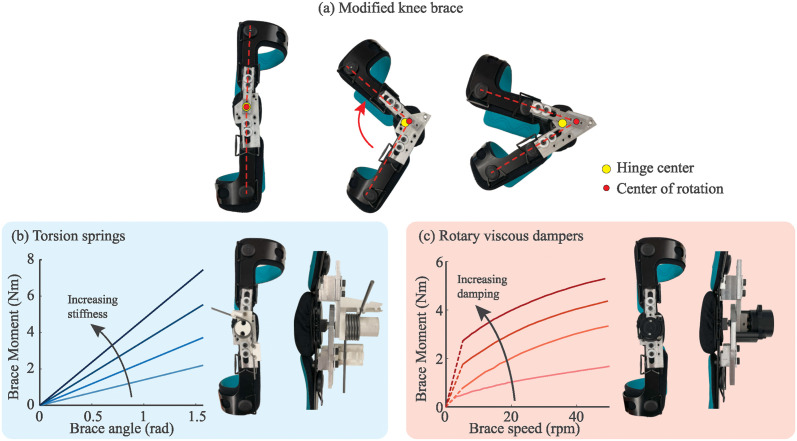
Modified knee brace used in the experiment. (a) Side view showing the modified joint-following mechanism; (b) implementation of torsion springs along with the selected moment-angle behavior of the four springs; (c) implementation of rotary dampers along with the moment-speed behavior of the four dampers used (data was imported from damper data sheets–dashed line indicates interpolated data).

We modified a commercial knee brace to serve as a reconfigurable knee exoskeleton with adjustable knee dynamics. We started with an Orthomen Functional Knee Brace (Orthomen, CA, USA) for the right leg. We then modified the brace to add a joint-center-following mechanism to serve as a rotary joint, see Fig 1a. Next, we added hardware features that allowed us to configure the knee to have different flexion stiffness or damping.

The Orthomen brace is a standard rigid knee brace with a gear-based polycentric hinge. We added two aluminum levers along a prismatic joint which can travel along the upper and lower legs of the brace, respectively. The ends of the two aluminum levers were connected using a rotary joint; the rotary joint was constrained to slide normal to the knee joint using a bronze sleeve bearing and a slot. This slot, along with the prismatic joints along the legs, enabled the aluminium levers to follow the instantaneous center of rotation of the brace. The aluminum plates included multiple tapped holes for mounting the springs and dampers.

We used swappable torsion springs and rotary dampers because the two parameters represent simple versions of two common sources of mechanical impedance found in exoskeletons. Springs and dampers may cause muscles to activate differently during walking [21]; therefore, we chose to investigate the effects of both types of impedance. While mass represents another common form of mechanical impedance, its effects on gait during walking [22–24] and running [19] are already fairly well characterized.

We used a set of 120 deg torsional springs with stiffness 1.15 Nm/rad and 2.37 Nm/rad (McMaster-Carr, IL, USA) to modulate the brace stiffness. The springs can be stacked in four combinations to achieve four different stiffness values. The total torsional stiffness settings of the device are 1.15, 2.37, 3.52, and 4.75 Nm/rad, see Fig 1b. The springs were mounted to the brace using 3D printed mounting brackets. Since the higher-stiffness springs are physically larger than the smaller springs, we concealed the springs during experimental trials by incorporating a panel to hide them from the subject’s line of sight.

We used a set of four different rotary viscous dampers, which operate using a vane submerged in viscous silicone oil to provide fluid resistance. The estimated maximum damping force of the dampers are 1 Nm, 2 Nm, 3 Nm, and 4 Nm at 50 RPM (FRT-F2 series; Bansbach Easylift, Lorch, Germany), see Fig 1c. The dampers display a slight nonlinear loading behavior, but we approximated the damping coefficients as 0.19, 0.38, 0.57, and 0.76 Nm/rad/s. The dampers were coupled to the knee brace using a 3D printed mount and a steel set-screw based shaft coupler. The dampers each have the same size, dimensions, and similar masses ranging from 78.3 g to 115.6 g. The dampers can be disengaged from the leg for the zero-stiffness case by removing the coupling plate. Because the dampers had the same dimensions and size, they were indistinguishable from each other while the brace was worn.

The knee brace and springs provide some nominal damping behavior from bearing or gear friction at the hinge. We experimentally measured the force-deflection of the brace at each stiffness setting and estimate a dissipation around 0.4—0.6 J of energy every stroke. Since this constitutes less than 25% of the energy storage at even the smallest stiffness configuration, we expect these additional friction sources to have a negligible effect on brace stiffness, damping, or the study results.

### Instrumentation and data processing

**Fig 2:**
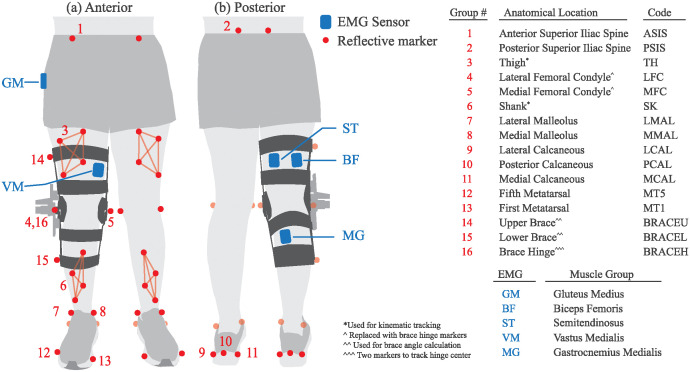
Visualization of the placement of retroreflective markers and EMG sensors on the body along with a table with the corresponding anatomical descriptions. Retroreflective markers were placed on anatomical landmarks of the lower limb and on the knee brace. Wireless surface EMG sensors were placed on the gluteus medius (GM), vastus medialis (VM), semitendinosus (ST), biceps femoris (BF), and medial gastrocnemius (MG). We added three additional markers to the rigid frame of the brace to measure brace deformation independently of biological knee angle.

Before each trial, retroreflective markers were affixed to the knee brace and the participant’s lower body, see Fig 2. Marker positions were recorded with a three-dimensional motion capture system (200 Hz; ten-camera system, Vicon, Oxford, UK) synchronous with ground reaction force (GRF) and electromyograph (EMG) data.

The level walking tasks were performed on a split-belt instrumented treadmill (2000 Hz; Bertec, Columbus, OH). The forces during the stair tasks were measured using six in-ground force plates (1000 Hz; AMTI, Watertown, MA, USA). GRF and synchronized motion capture data were filtered with a zero-phase, 4th-order, low-pass Butterworth filter with a cutoff frequency of 15 and 8 Hz, respectively. Motion capture data and GRF data were processed in Visual3D (C-motion, Germantown, MD, USA) to derive sagittal lower limb joint angles, speeds, and moments.

Muscle activity was measured using five wireless surface electromyography sensors with 10 mm electrode distance (2000 Hz; Delsys, Natick, MA, USA). The EMG signals were initially filtered using a 100 Hz high-pass Butterworth filter [25]; 100 Hz was chosen as it heavily reduces movement artifacts without affecting the shape or relationship of muscle activation [26, 27]. The envelope of the signal was subsequently estimated using a moving root-mean-square (RMS) filter with a window size of 0.125 seconds. Movement artifacts were further highlighted and removed by finding and removing RMS values with a magnitude three standard deviations higher than the data distribution for a given percentage of gait. The sensors were placed on knee flexor muscles including the biceps femoris (BF), semitendinosus (ST), medial gastrocnemius (MG); one knee extensor muscle, the vastus medialis (VM); and a hip adductor muscle, the gluteus medius (GM). All sensor preparation and locations were placed based upon recommendations from the SENIAM project [28]. Electromyograph data were normalized to the average RMS values recorded during the control trials for each participant.

Perception of being impeded was recorded using a series of questions with a 7-point Likert scale. After every experimental condition, participants were asked to rate from “strongly disagree” to “strongly agree” their agreement to the statement “I feel impeded by the knee brace during this activity”, where “this activity” was replaced with “walking”, “stair ascent”, or “stair descent”. Initially, the participants were shown a neutral or unsure option (4/7), but if they selected this for a task, then they were subsequently asked to choose between “slightly agree” or “slightly disagree”.

Custom MATLAB scripts (Mathworks, Natick, MA, USA) were used for subsequent data processing and analysis. Gait strides were normalized in length and split into individual strides. Toe clearance was calculated by finding the minimum vertical height of the toe markers during swing, relative to the vertical position of standing [29]. Brace angle was calculated using the hinge marker locations and the upper and lower brace marker locations.

Transparency thresholds were determined by using two criteria: (i) changes in perception and (ii) changes in ground clearance. Perception thresholds were determined by finding the stiffness or damping value at which a given participant switched from feeling unimpeded to feeling impeded by the device. This estimation was separately performed for both stiffness and damping and for walking, stair ascent, and stair descent. If a participant did not feel impeded for any given trial, then we did not determine a threshold for that individual and condition.

Ground clearance thresholds were determined from the distribution of minimum toe clearance data for each participant across the last 25 strides of each trial (to account for any possible adaptation effects during the first ∼50 strides). According to [30], the distribution of minimum toe clearance can be modeled to estimate the probability of an individual stride falling below a specified height. Typically, the kurtosis and skewness of the distribution play key roles in estimating toe scuffs below 0.5 cm, but around 1 cm, a normal distribution is similarly accurate. Therefore, given the limited number of strides per trial, we fit a normal distribution to each participant’s minimum toe clearance data to estimate the likelihood of scuffing an unseen object of 1 cm in height. We then define a transparency threshold based on the stiffness or damping value at which the probability of scuffing exceeds a specific amount. Since the brace alone likely affects the probability of scuffing due to added mass and bulk, we subtracted all scuffing probabilities from the scuffing probability with the brace alone. For this study, we use a 20% probability to determine ground clearance thresholds as it captured changes across the greatest number of participants. This procedure using toe clearance was done for level walking only. To confirm that minimum toe clearance was a reasonable metric to use for defining a threshold, we performed a statistical analysis on this metric to ensure toe clearance changed as a function of stiffness and damping. Specifically, we performed a repeated measures ANOVA with *α* = 0.05 and a Holm-Sidak correction. We did not determine a toe clearance threshold for stair navigation as there is no existing literature mapping the probability of scuffing or unsuccessful strides to variations of toe clearance.

### Experimental protocol

**Fig 3:**
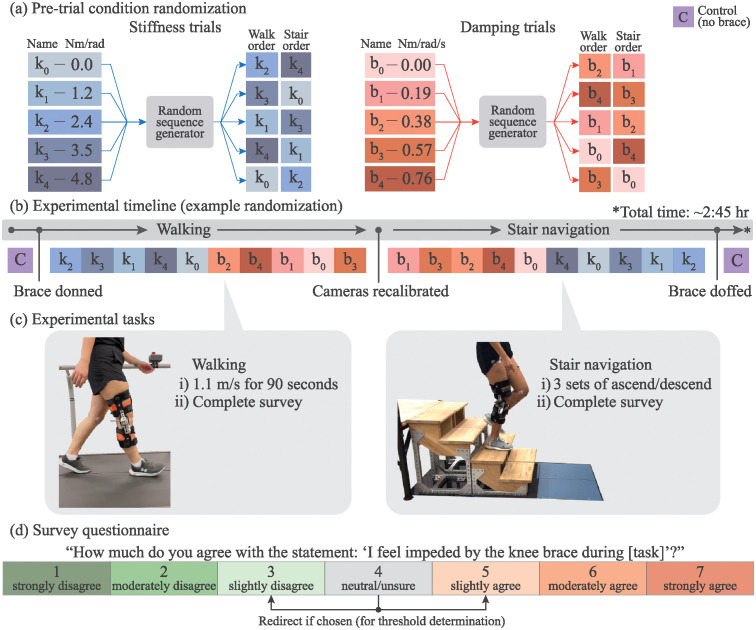
Experimental organization. (a) Stiffness and damping randomization procedure. Before each experiment, the stiffness values and damping values are shuffled and randomly assigned to the trial number for both walking and stair trials; (b) the order of experimental conditions completed, where C is the control with no brace, k represents stiffness trials, and b represents damping trials; (c) the walking and stair navigation tasks for each corresponding experiment grouping. The walking tasks and stair tasks were performed sequentially to allow re-calibration of the motion capture cameras for each respective task; (d) the survey questionnaire asked after each trial.

Data collection occurred in one experimental session for each participant, see Fig 3. At the beginning of each session, the brace was briefly donned where the hinge of the brace was aligned with the biological knee joint and the padding was adjusted for comfort. The participants were instructed to navigate stairs and walk around the room to check for brace alignment, comfort, and brace migration; subsequent adjustments were made. After marking the position of the brace on the leg, the brace was doffed. A total of 38 retroreflective markers were placed on lower-limb anatomical landmarks, see Fig 2. The six EMG sensors were affixed to the respective muscle at the closest location to the belly of the muscle between straps of the brace.

The experiment occurred in two main sections: walking and stair navigation. Walking was chosen as the primary task as it is the most common daily gait task; stair ambulation was chosen as a secondary task as it requires much larger range of motion from the knee (up to 90 deg during swing compared to 70 deg during walking [31]) and also uses the knee to supply power or dissipate power during stair ascent and descent, respectively [32]. The order of stiffness and damping values for each trial were randomized to minimize confounding effects from neuromechanical adaptation.

A control activity was performed first, where the participant was instructed to walk for 90 seconds at 1.1 m/s without the brace. We use 90 seconds for each walking trial to ensure that each participant had adequate time to adapt and stabilize to the changes in lower-limb mechanical properties [18]. Due to the various knee brace conditions after the control trial, the two right knee markers were removed and the brace was carefully placed back at the original marked location. The first randomized stiffness condition was then affixed to the brace and the participant was instructed to walk for another 90 seconds. The participant was prevented from seeing the springs being placed. After the trial, the participant was asked the survey question whether they felt impeded by the brace.

This procedure was repeated for the remainder of the randomized set of stiffness values and then the set of randomized damping values. The stiffness trials and damping trials were performed in blocks due to time constraints, as switching from the damper mounts to spring mounts takes significantly longer than switching within each type.

The stair trials began after the walking trials were completed. Since the stairs were mounted to the in-ground force plates, the motion capture cameras were re-calibrated to the location of the stairs. The stair navigation tasks used a set of four identical staircases (width: 405 mm, depth: 250 mm, rise: 175 mm), with two steps each, and were placed on top of four force plates, see Fig 3c. We included an elevated platform after the tallest set of stairs such that participants could continue walking once reaching the top.

The stair trials began with a set of re-randomized damping conditions and then proceeded to a set of re-randomized stiffness conditions. The participant was asked to lead (i.e., take their first step) with their left leg every trial and perform step-over-step stair ambulation. The participants were instructed to ascend then descend the stairs three times, and were subsequently asked separate ascent and descent survey questions after each set.

At the end of the final stiffness trial, the brace was doffed for the final time and the right knee markers were re-affixed. The participant was instructed to complete a set of stair navigation without the brace as a final control condition.

## Results

### Walking kinematics

**Fig 4:**
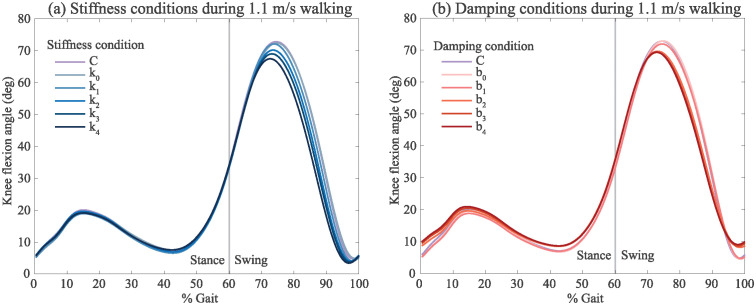
Group mean knee flexion angle during 1.1 m/s walking (N = 10) for the (a) stiffness and (b) damping conditions, normalized to percent gait. A separating line between stance phase and swing phase is drawn at 60% gait.

**Fig 5:**
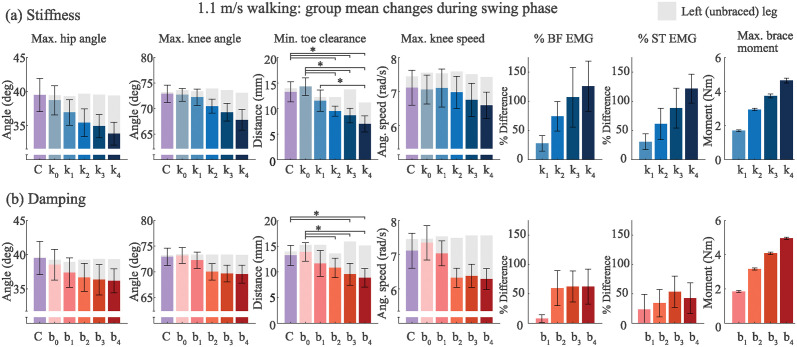
Group mean results for 1.1 m/s walking experiment (N = 10) for (a) stiffness and (b) damping conditions. Kinematic variables including maximum hip flexion angle, maximum knee flexion angle, minimum toe clearance, and maximum knee speed are displayed for every experimental condition during swing phase. Change in peak swing phase muscle activity from the brace-only condition (e.g., *k*_0_, *b*_0_) are shown for the biceps femoris (BF) and semitendinosus (ST) muscles. Finally, peak brace moment during swing phase for each condition is shown. The error bars represent one standard deviation (*p < 0.05, shown for minimum toe clearance variable only).

We observed that as stiffness and damping increased, the average peak knee flexion angle during swing decreased (Figs 4 and 5). Other kinematic variables, namely minimum toe clearance and maximum hip flexion, also generally decreased with higher knee stiffness during swing phase (Fig 5a). From control to maximum stiffness *k*_4_, we observe a decrease in average peak swing knee flexion angle from 72.9 deg to 67.8 deg. Increasing stiffness also resulted in a decrease of group mean hip flexion angle from 39.5 deg to 33.8 deg. Increasing damping caused a small decrease to knee flexion angle and hip flexion angle.

As stiffness increased, the group mean minimum toe clearance significantly reduced from 14 mm at *k*_0_ to 7 mm at *k*_4_ (*p* < .001) (Fig 5a). Likewise, as damping increased, the mean minimum toe clearance significantly reduced from 14 mm at *b*_0_ to 9 mm at *b*_4_ (*p* < .001) (Fig 5b). Previous studies reported a range of minimum toe clearance during normal treadmill walking of 15 ± 4 mm [33] and 14.9 ± 2.8 mm [34], resulting in a 7.1 and 6.4% error of the median, respectively, between the mentioned studies and our control condition.

As damping increased, the peak hip flexion angle, peak knee flexion angle, and toe clearance slightly decreased on average. Mean maximum knee speed decreased from 7.5 rad/s to 6.2 rad/s in the maximum damping condition compared to the *b*_0_ condition.

Additionally, we measured stride frequency and stride length; however, no changes between any of the experimental conditions were found.

### Walking EMG

Hamstring muscle activity during swing phase generally increased as a function of stiffness. The group mean biceps femoris peak RMS muscle activity increased by 125.9% and the semitendinosus by 121.7% from the zero-stiffness brace condition *k*_0_. Additionally, there were no measurable changes to the gluteus medius or vastus medialis muscle activity among conditions. Despite a reduction in knee flexion angle, a strong correlation existed (*r* = 1.00) between the brace moment and brace stiffness, increasing linearly from 2 Nm in *k*_1_ to 5 Nm in *k*_4_.

The biceps femoris showed an increase in muscle activity starting with *b*_2_ at 59.6%, but the average increase in muscle activity did not surpass 62.1% as damping values increased.

### Stair kinematics

**Fig 6:**
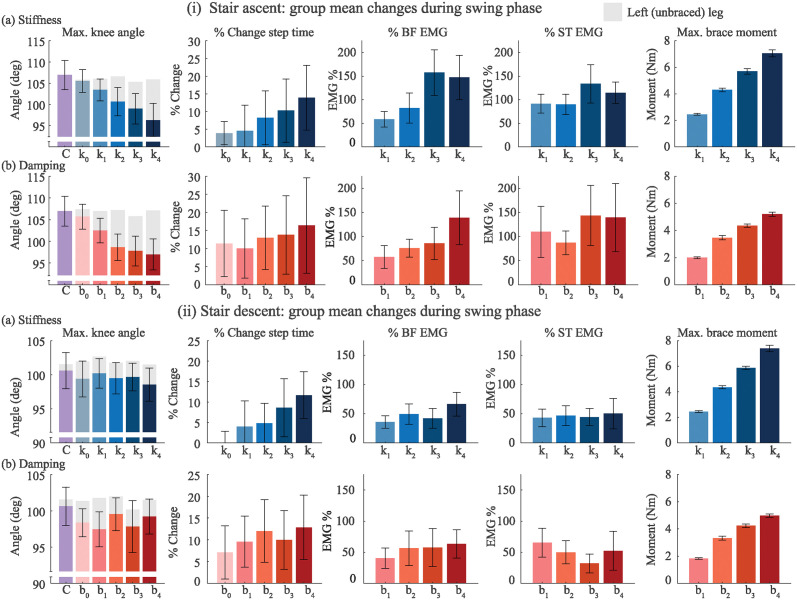
Group mean results for (i) stair ascent and (ii) stair descent tasks (N = 10) for each (a) stiffness and (b) damping conditions. Kinematic variables including maximum knee flexion angle and change in step time from control for each experimental condition during swing phase. Change in muscle activity from the control condition (e.g., *k*_0_, *b*_0_) during swing phase are shown for the biceps femoris (BF) and semitendinosus (ST) muscles. Finally, the peak brace moment for each stiffness condition during swing phase is shown. The error bars represent one standard deviation.

In stair ascent, similar to walking, we observe a decrease to the peak knee flexion angle as brace stiffness was increased (Fig 6i). The peak knee flexion reduced on average from 107.0 deg in the control to 96.3 deg in *k*_4_. Similar to walking, the range of motion or knee velocity did not show any trends. We observed a small trend with mean step time (time taken to traverse two steps) increasing as a function of stiffness. Damping also affected the peak knee flexion angle, reducing from an average of 107.0 deg in the control to 97.0 deg in *b*_4_ (Fig 6ii). Stair ascent also showed a highly variable increase in step time, indicating a difference in reactions of participants–some of whom walked at slower speed with the dampers, while others maintained their nominal speed.

During stair descent, there were no trends with peak swing knee flexion angle during swing phase in any conditions. We observed that the time taken to descend was slightly slower in both conditions, with the maximum stiffness taking 13% longer than the control and the maximum damper taking 15% longer.

### Stair EMG

Both stair ascent and stair descent resulted in higher hamstring muscle activity during swing phase as stiffness and damping increased (Fig 6ii). During stair ascent, the biceps femoris muscle showed a mean peak swing increase from the *k*_0_ to the *k*_4_ condition of 157.7%, while the semitendinosus showed an increase of 133.7%. Damping also generally increased peak hamstring activity; the biceps femoris and semitendinosis showed swing phase increases at *b*4 of 138.8% and 143.6%, respectively. During stair descent, there was a slight increase in peak swing hamstring EMG in all cases. The maximum stiffness *k*_4_ increased the mean peak swing biceps femoris activity by 66% and the semitendinosus by 50%, while the maximum damping *b*_4_ resulted in a mean increase of 63% and 52% in the biceps femoris and semitendinosus, respectively. The vastus medialis, gastrocnnemius medialis, and gluteus medius did not show measurable changes during both stair ascent and descent.

During stair ascent and descent, the brace applied a mean peak moment of approximately 2.3 Nm in the *k*_0_ and *b*_0_ conditions up to 6.2 Nm during the *k*_4_ and *b*_4_ conditions.

### Perception of being impeded

**Fig 7:**
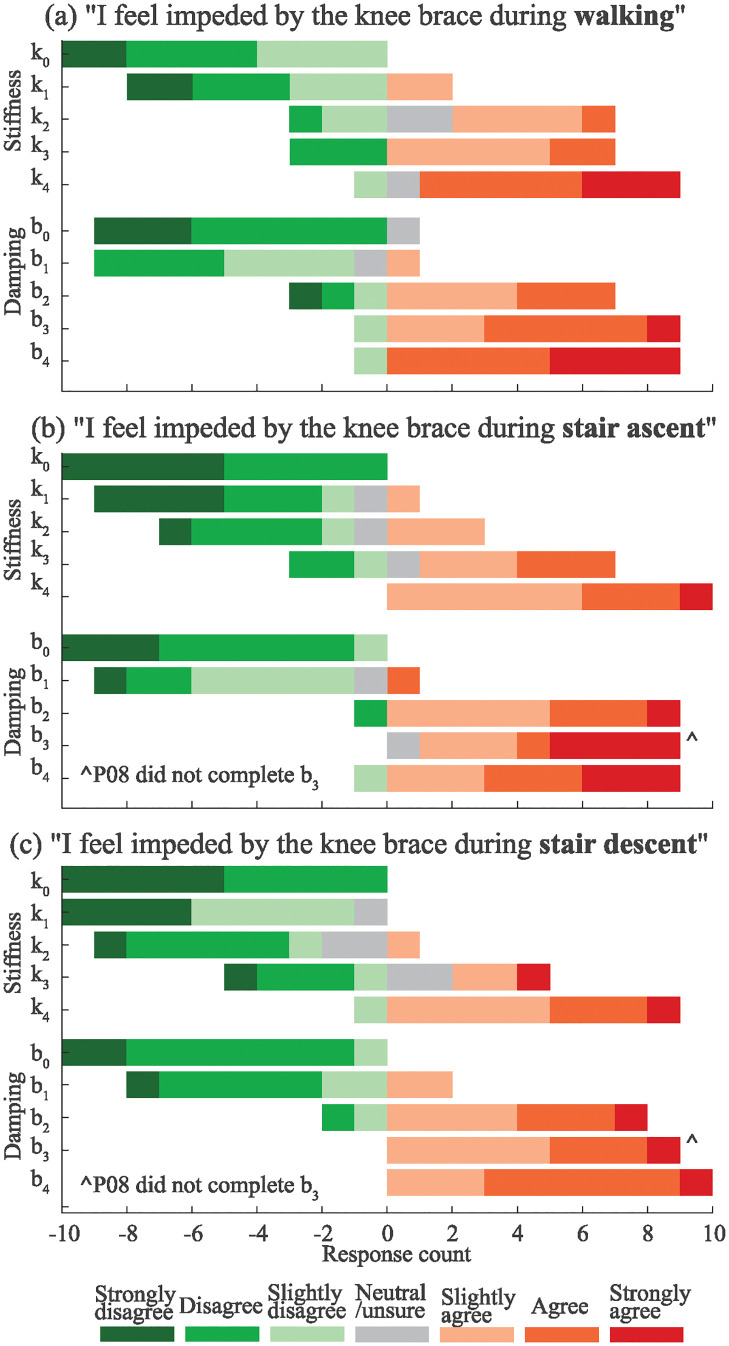
Group survey results for different stiffness and damping conditions (N = 10). (a) Responses after 1.1 m/s walking, (b) responses after stair ascent, (c) responses after stair descent.

As stiffness or damping increased, the perception of being impeded increased across all conditions, see Fig 7. The left and right sides of the plot represent the number of responses considered “not impeded” and “impeded”, respectively; neutral responses were placed on the side the participants aligned with closer based on the forced-choice question. During walking, the number of participants who reported they felt impeded by the brace linearly increased with the stiffness magnitude, crossing to majority feeling impeded between *k*_1_ (1.15 Nm/rad) and *k*_2_ (2.37 Nm/rad). As damping increased during walking, the survey responses sharply switched from positive to negative between *b*_1_ (0.19 Nm/rad/s) and *b*_2_ (0.38 Nm/rad/s).

During stair ascent, the same linear relation existed with stiffness, but the linear relation shifted such that the majority initially felt impeded between *k*_2_ (2.37 Nm/rad) and *k*_3_ (3.52 Nm/rad). As damping increased, a similar relation was observed as in walking, where the opinion switched quickly from positive to negative between *b*_1_ (0.19 Nm/rad/s) and *b*_2_ (0.38 Nm/rad/s), We note that for both stair ascent and stair descent, participant P08 did not complete the third damping condition due to a mechanical issue with the brace.

For stair descent, the majority switched to feeling impeded between *k*_2_ (2.37 Nm/rad) and *k*_3_ (3.52 Nm/rad) and between *b*_1_ (0.19 Nm/rad/s) and *b*_2_ (0.38 Nm/rad/s).

### Transparency thresholds for stiffness and damping

**Fig 8:**
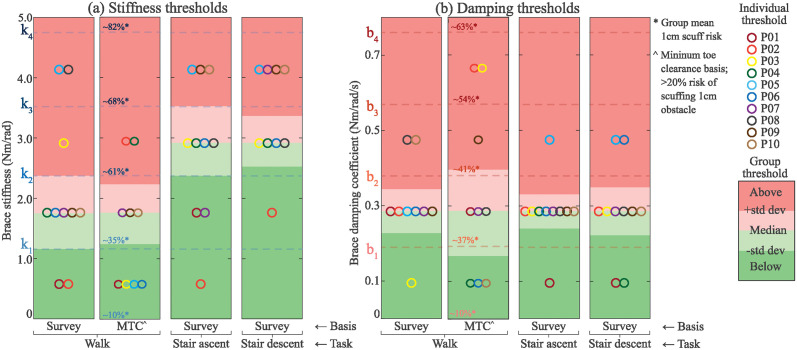
(a) Stiffness thresholds and (b) damping thresholds, separated by activity and basis of threshold. Individual thresholds (circles) and group thresholds (colors) are shown. Survey based thresholds are defined as the amount of stiffness or damping at which each participant’s survey response crossed from “disagree” to “agree”. Minimum toe clearance (MTC) based thresholds are defined as the stiffness or damping at which the risk of scuffing an unseen 1 cm tall obstacle [30] exceeds 20% per stride. The group mean risk of scuffing an unseen 1 cm object is shown at each stiffness value. Participants were excluded if no threshold could be determined.

Fig 8a shows the perceived stiffness and damping thresholds we determined for the group median and for each participant. The stiffness threshold during walking had a median value of 1.76 Nm/rad while the median threshold was 2.94 Nm/rad in stair ascent and stair descent. There was moderate variability between perceived stiffness thresholds, with the range of individual responses covering the whole stiffness range (0.5 Nm/rad to 4.13 Nm/rad).

The perceived damping thresholds for walking, stair ascent, and stair descent all had a group median threshold of 0.285 Nm/rad/s.

Fig 8b shows the stiffness and damping thresholds based on toe clearance during walking. This chart displays the group median as well as individual threshold stiffness and damping values at which the chance of scuffing a 1 cm tall object exceeded 20%. We excluded one participant as their toe clearance did not significantly change between conditions.

## Discussion

The aim of this study was to characterize the effects of stiffness and damping applied at the knee joint to better understand perception and biomechanical responses in order to inform exoskeleton design. Perception, kinematics, and muscle activity showed significant but varied (participant-specific) changes in response to higher stiffness and damping.

We leveraged user perception of being impeded and toe clearance to determine transparency thresholds, below which participants were sufficiently unimpeded. For knee stiffness, we found the median transparency thresholds for walking and stairs to be 1.76 Nm/rad and 2.95 Nm/rad, respectively. For knee damping, we found the median transparency threshold for walking and stairs to be about the same, 0.29 Nm/rad/s.

Using individual data, we then produced a distribution of transparency thresholds in terms of stiffness and damping. Several additional trends were observed that may help exoskeleton designers better understand and prepare for potential reactions to impedance sources as the knee during leg swing, which we discuss in the following section. We also discuss limitations of the present study and other broader implications and considerations.

### Individual variability

Each participant demonstrated variable reactions to increasing knee stiffness and damping during swing. There are likely at least two mechanisms responsible for this variability: (i) randomization order, and (ii) different compensatory mechanisms. Randomization order has a potential impact on user variability since the relative changes of stiffness and damping were more easily perceived when the differences were high relative to the preceding condition. For example, if switching from a large stiffness to a lower stiffness (e.g., *k*_5_ to *k*_3_), a participant may perceive that the brace no longer impedes them at *k*_3_; whereas the participant may feel more impeded when they go from a stiffness of *k*_1_ to *k*_3_.

Another prominent reason for inter-subject variability is that individuals could use different compensation strategies. For instance, a person could increase their hamstring muscle activity during swing phase to overcome higher impedance, or they could flex their knee less when impeded, or they could do a combination of each of these. Across our participant sample, we observed individuals who used each of these strategies. Within a single participant, we even observed indications of different strategies being used for different locomotor tasks or amounts of impedance.

**Fig 9:**
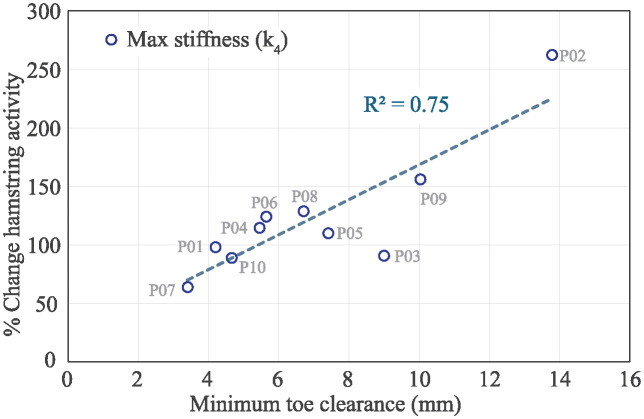
Relationship between change in hamstring muscle activity (averaged BF and ST percent) and mean minimum toe clearance for each participant during the maximum stiffness condition.

Generally, we observed a trade-off between hamstring muscle activity and kinematics during swing phase. For example, P01 significantly decreased minimum toe clearance from 15.0 mm to 4.2 mm, and demonstrated an increase in peak swing semitendinosus (ST) activity of 118%. On the other hand, P09 only slightly decreased mean toe clearance from 13.6 mm to 10.0 mm while they increased peak semitendinosus activity by much more, 226%. The former participant may have been trying to minimize muscle exertion at the expense of potentially scuffing the ground due to low foot clearance, while the latter participant may have been trying to maintain kinematics or foot clearance but at the cost of their muscles working harder against the impedance. While there were not enough participants to make broader claims about the neuromechanics of compensatory strategies, we do observe correlations within our study population that demonstrated this muscle-activity and kinematics trade-off, see Fig 9. In this figure, we plotted the mean change in muscle activity of the hamstring muscles (mean percent change of BF and ST) versus the minimum toe clearance at the maximum stiffness condition. The results demonstrated a positive correlation between the two variables (*R*^2^ > 0.7). The same trend was also present for the maximum damping condition, indicating the trade-off may occur under both impeding conditions.

### Comparing stiffness and damping

While springs and dampers both reduced motion of the knee and increased knee flexor muscle activity, there were a few qualitative differences between the two impedance sources. These suggest that dampers and springs may need somewhat different considerations from a human-factors design perspective.

Survey responses both trended consistently towards more impeding as spring stiffness or damping coefficient increased. However, there are a few subtle differences, especially during stairs. Survey responses for damping conditions were generally stronger than the corresponding stiffness conditions during stair ascent, despite the brace moment from springs being higher during stair navigation (e.g., during stair ascent, 90% of participants found *b*_2_ to be impeding, while only 30% of participants found *k*_2_ to be impeding), see Fig 7. Damping had the same thresholds for all conditions while stiffness was generally less subjectively impeding during stairs. One explanation could be the purely dissipative nature of dampers compared to springs. Since springs return the energy that has been stored, this may improve favorable user-perception as the energy is returned. The drastic shift in perceived impedance could also be due to the non-linearity of the dampers (see Fig 1).

During stair navigation, the dampers applied a lower peak moment (5.0 Nm in *b*_4_) than the springs (7.4 Nm in *k*_4_) because knee flexion angle increased significantly but knee velocity did not increase significantly. More research is needed into the effect of dampers on self-controlled walking, as one compensatory strategy may be to simply slow down. The speed-dependent nature of damping vs. the position-dependent nature of stiffness may play a key role in the tolerable amounts of damping or stiffness for a given activity.

During walking, kinematic and muscle activity changes were mostly linear with respect to stiffness, whereas in the dampers, the kinematics and muscle activity tended to level out. This is perhaps because the dampers both reduced maximum knee flexion and increased minimum knee flexion (Fig 4), whereas springs tended to reduce both. This is especially apparent during stair descent, where dampers increased the minimum knee flexion from 11.5 deg in the control to 17 deg in *b*_4_, while the springs reduced minimum knee flexion to 8 deg in *k*_4_.

While springs and dampers are representative of simple sources of mechanical impedance, designers must carefully consider all forms of mechanical impedance beyond what was evaluated in this work. For robotic exoskeletons, common forms of impedance are blacklash from gearing, reflected inertia/damping, and the load-dependent transmission losses from various gearing types [35]. Passive exoskeletons that utilize springs may not necessarily demonstrate linear behavior, or in the case of composites such as rubber, the springs may act partially as dampers [36]. Additionally, passive mechanisms such as clutches may employ constant-force springs which in turn apply constant forces to the knee [37]. Combinations of spring equilibrium and stiffness have been shown to impact walking performance [38], and may have different effects on the response to or perception of stiffness.

Impedance may also be time or environment dependent, as in the case of viscous dampers, where a change in ambient temperature results in a subsequent change of the damping coefficient. Designers would need to consider the range of environmental conditions when creating a device to ensure it meets a target transparency threshold during typical use cases. Real-world braces, orthoses, and exoskeletons may apply any combination of linear or nonlinear impedance to the knee, and more research is needed into specific responses to combinations of impedance. Future studies could aim to establish the transparency thresholds for combinations of stiffness and damping. However, our results provide initial guidance for designers to estimate a baseline stiffness or damping threshold, or an equivalent knee torque. These results (Figs 5 and 6) provide a point of reference, which is an improvement to having no quantitative reference at all.

### Thresholds for muscle activity

While we observed a large change in peak swing-phase hamstring muscle activity relative to the control condition, we did not present design thresholds based on muscle activity. This is primarily because it was unclear how to objectively define a threshold. On average, increases in EMG were fairly linear, particularly during walking (Fig 5). Furthermore, while the percentage change in muscle activity is relatively high, exceeding 100%, the RMS values experienced during swing did not exceed the RMS EMG values experienced during stance phase in the control condition. We observed a peak mean swing EMG of 13% of the peak stance EMG at the highest stiffness condition *k*_4_, and a peak mean swing EMG of 6.4% of the peak mean stance EMG with *b*_4_. As a consequence, it is difficult to know where to draw the transparency threshold in terms of EMG increases. How much EMG increase is too much is often subjective, ill-defined, or not generalizable. Instead, we found ourselves consistently going back to the user perception or foot clearance data to define meaningful transparency thresholds.

### Device performance

The brace served as an adequate, relatively lightweight, and simple experimental platform to provide moments about the knee joint. Participants did not report any backlash or excess resistance from the mechanism we added to the brace. While the brace did not migrate during trials significantly, comfort notably decreased near the end of the experimental sessions. The compression of the leg with the brace straps caused slight discomfort and sweating in some participants. However, most knee exoskeletons interact with the knee in a similar manner, and no practical alternatives exist for transferring force to the leg within the experiment. Randomization in the study design helped mitigate these effects on the study results.

### Study limitations

One of the primary limitations of this work is the resolution of stiffness and damping tested, which was only able to give an estimate of threshold stiffness with a resolution of ±1 Nm/rad and threshold damping with a resolution of 0.2 Nm/rad/s, approximately 1 Nm moment for both. Also, only stiffness and damping were independently tested. Our results may provide baseline approximation, but may not directly apply to other impedance sources (e.g., reflected inertia, viscoelastic materials).

Another limitation is that this brace was only tested at a single walking speed on consistent terrain. While we use walking and stair navigation to obtain a nominal transparency threshold, these results may not be fully translatable to faster walking speeds or running. A follow-on study would be needed to empirically test the tolerance of impedance across locomotion types and speeds, or when navigating uneven terrain. However, until further activity-specific research is completed, the transparency thresholds identified in this current study provide a reasonable starting point for designers. During the stair navigation trials, the small number of steps may also affect the results as there is little time for transient effects to stabilize. The study was also performed in one experimental session with each condition/activity being performed once due to time constraints and potential effects from fatigue.

Additionally, the brace interfered with the optimal placement of the vastus medialis and gastrocnemius medialis EMG sensors; future studies may need to consider thinner electrodes that may go under the straps. While we sought to explore the effects of stiffness and damping on swing phase, our knee brace affected both stance and swing phase. However, since the moments applied by the knee brace were small in stance (< 2.5 Nm in walking) relative to the magnitude of biological knee moments during stance (> 45 Nm in walking), but were a significant portion of biological swing moments (often exceeding 20%), we considered this a reasonable experimental means to explore the impacts on swing phase.

Survey responses are a source of bias as well, since participants were making decisions based on constrained choices, the scaling of which can vary significantly depending on the range of stimuli tested [39]. The survey responses may not represent the true perception felt in the context of using a real exoskeleton, where they can internally weigh the benefits versus the impedance provided by the device. Adaptation likely plays a role in the perception of stiffness or damping over time, especially as users familiarize with the device. Over time, this may shift the transparency thresholds higher—however, biomechanical markers such as muscle activity would increase. More investigation is needed into long-term responses to exoskeletal impedance.

Finally, this study was only performed on a convenience sample of generally young, non-clinical populations. Statistical comparisons would be underpowered for the number of conditions and variables reported, which is why we only performed statistical analysis on the minimum toe clearance variables used to define the transparency threshold. For all other outcome metrics, the main value of this study is in elucidating trends and average magnitudes of changes. Populations suffering from knee injuries, knee diseases, or elderly populations may have different tolerances for impedance at the knee, and may need to be tested or considered separately. For instance, elderly populations tend to have higher risks of scuffing the ground due to higher variability in toe clearance [33, 40]. This group would likely have lower toe clearance stiffness thresholds, and as a consequence may have lower transparency thresholds.

## Conclusion

Stiffness and damping applied at the knee joint resulted in biomechanical and perceived consequences to gait, especially above a stiffness of 1.76 Nm/rad or damping coefficient of 0.29 Nm/rad/s. Biomechanical metrics varied between participants but showed participants face trade-offs between minimizing muscle activity and maintaining kinematics. One of the main biomechanical consequences was reduced toe clearance, which increases the likelihood of scuffs and tripping. By considering these effects, exoskeleton designers can use the thresholds determined in this study to set design constraints on knee stiffness and damping to minimize feelings of being impeded or the likelihood of foot scuffing. Based on our data, we recommend that designers try not to exceed a knee stiffness of 1.76 Nm/rad or a damping of 0.29 Nm/rad/s, both of which correspond to peak moments during swing phase of 2.3 Nm. When exceeding these thresholds during walking, users may feel impeded by exoskeleton or have a significantly higher chance of scuffing the ground.
